# The bacteriological quality of different brands of bottled water available to consumers in Ile-Ife, south-western Nigeria

**DOI:** 10.1186/1756-0500-7-859

**Published:** 2014-11-28

**Authors:** Oluwatoyin A Igbeneghu, Adebayo Lamikanra

**Affiliations:** Department of Pharmaceutics, Obafemi Awolowo University, Ile-Ife, Nigeria

**Keywords:** Bottled water, Water quality, Coliforms, Indicator organisms, Water bottling companies

## Abstract

**Background:**

The upsurge in the demand for bottled water has prompted the interest of many manufacturers in the production of bottled water and very many water bottling companies are therefore involved in its production. These range from large scale multinational companies to medium scale business enterprises, institutional and government business investment companies as well as small scale entrepreneurs. There is however little information on the comparative quality of bottled water brands produced by different classes of water bottling companies in Nigeria. This study was undertaken to determine the bacteriological quality of brands of bottled water available to consumers in Ile-Ife.

**Methods:**

Forty-three samples of bottled water comprising of three batches each of thirteen bottled water brands and two batches of two brands were purchased and analyzed for total bacterial count, presence of coliform and the presence of other bacterial indicators of drinking water quality.

**Results:**

Only 67.4% of the water samples representing the products of 10 companies or 66.7% of the brands had heterotrophic counts within the acceptable limits. Coliforms present in 100 ml of water were detected in 26.7% of the bottled water brands. Other indicator organisms detected included Staphylococci isolated from 27.9% of the samples (33.3% of the brands) and specifically *Staphylococcus aureus* found in four brands constituting 14% of the samples. *Pseudomonas* strains were consistently detected in consecutive batches of three brands of the water samples.

**Conclusions:**

Bottled water samples produced by the large scale multinational producers were of acceptable bacteriological quality unlike those produced by most small companies. Significance and Impact of Study: There is need for a greater control of water bottling processes carried out by commercial bottled water producers in Nigeria.

## Background

Water is indispensible to life as it is required for all physiological processes which demand that all living organisms have ready access to water. This need is no less important to human beings who have to drink plenty of water every day [1]. Apart from drinking, man uses water for many domestic, industrial and recreational purposes which include washing, bathing, cooking, food processing, brewing and beverage bottling as well as sporting activities. This means that there is a need for the constant supply of potable water to all human communities and in areas where such supplies are lacking, a great deal of time and effort are devoted to finding a suitable source of supply. Unfortunately, such water sources even when they are available, are seldom safe or reliable and waters obtained there from need to be treated appropriately in order to make them potable.

In all urban areas in the developed countries, reliance is placed on the supply of adequately treated water by municipal authorities. In developing countries however there is little or no access to such treated water and so, potable water is usually difficult or even impossible to get. This is because very little money has been made available for the appropriate municipal infrastructure and this being so, a large percentage of people in these countries have to depend on their own individual efforts to get the water which they need. One of the means of satisfying the need for potable water especially in urban communities is to consume packaged water which in Nigeria is sold in plastic sachets called pure water or in plastic bottles [2]. Bottled water is drinking water which has been packaged in plastic bottles ranging in size from small single serving polyethylene terephthalate bottles of 500 ml −1.5 L capacity to large carboys (20 L) for water coolers [3]. In Nigeria, bottled water is regarded as being safer than water dispensed and sold in sachets, but it is also about ten times more expensive which is why it is patronized mainly by people with a relatively large disposable income.

Apart from microbiological considerations, the upsurge in the demand for bottled water has prompted the interest of many manufacturers in the production of bottled water. Close to two decades ago, bottled water was a product of a few multinational and large scale food processing and beverage producing companies in Nigeria. Presently however, there is the involvement of very many water bottling companies ranging from large scale multinational companies to medium scale business enterprises, institutional and government business investment companies as well as small scale entrepreneurs [4]. These water bottling companies use various water purification methods which may be one of or a combination of two of filtration, ozonisation, ultra violet irradiation and chlorination.

Bottled water has been reported to be associated with outbreaks of infections in the last few years. In 2006, *Salmonella enterica* serovar Kottbus from bottled water was significantly associated with 41 cases in an outbreak in infants in Gran Canaria. Nineteen of the cases had underlying disease or were immuno-compromised. The organism was isolated from bottled water randomly selected from the markets and in the local factory where the water was bottled [5]. Eckmanns *et al.* (2008) have described an outbreak of hospital-acquired *P. aeruginosa* infection caused by contaminated bottled water in intensive care units in a hospital in Germany [6]. In the hospital, the bottled water was used for the preparation of orally administered medications and oral fluid replacement. Some unopened bottles of water were found to contain the outbreak strain of *P. aeruginosa.* Cases such as these underscore the need for continual surveillance or monitoring of quality of bottled water. Studies on the quality of bottled water in many parts of the world including Canada, South Africa, Iran, Egypt and Nigeria have shown that bottled water samples are not always of the required microbiological quality [7–15]. This study was therefore carried out to determine the bacteriological quality of all available brands of bottled water sold in a popular shopping centre in Ile-Ife, an urban settlement which is home to both the Obafemi Awolowo University and the Obafemi Awolowo University Teaching Hospitals Complex as well as many commercial banks within which a large number of potential users of bottled water work. This study reports the comparative quality of bottled water brands produced by different classes of water bottling companies in south-western Nigeria.

## Methods

### Sampling

A total of 43 bottled water samples representing three batches of 13 brands of bottled water and two batches of two brands purchased on three different occasions within a period of 6 months were tested for bacteriological quality. On the first sampling occasion, samples of all brands of bottled water available for sale in shops located in Mayfair Hotel Shopping centre in Ile-Ife were purchased. The samples were inspected and ascertained to be in good condition with the caps and protective seal intact before purchase. The dates of production as well as the batch numbers were documented. They were taken to the laboratory and analyzed for total bacterial load and the presence of bacterial indicators of drinking water quality.

The sampling was repeated two more times at intervals of 3 months to include representative samples of three different batches of each brand of bottled water available for sale in Ile-Ife. On the third sampling occasion two of the brands were not available and so only two batches of these brands were represented in the study.

The brands were coded A-O for the purpose of this study and also categorized into three groups based on the size or class of the companies producing the water as shown in Table 1.

**Table 1:** **Tested brands of bottled water samples produced by different classes of companies**

Producers	Brand	Location of production plant	No. of batches	Method of disinfection
Multinational companies	A	Ibadan	3	Chlorination
	B	Lagos	3	Chlorination
	C	Lagos	3	Filtration and UV
Medium scale and institutional bottling companies	D	Ibadan	3	Chlorination
	E	Ile-Ife	3	Ozonisation
	F	Osogbo	3	Filtration and ozonisation
Small scale business entrepreneurs	G	Ile-Ife	2	Filtration and ozonisation
	H	Ile-Ife	2	Unspecified
	I	Ile-Ife	3	Filtration and ozonisation
	J	Ile-Ife	3	Unspecified
	K	Ile-Ife	3	Unspecified
	L	Ile-Ife	3	Chlorination
	M	Ile-Ife	3	Chlorination
	N	Ile-Ife	3	Ozonisation
	O	Ilesa	3	Filtration

### Test for the presence of chlorine in the samples

The caps and necks of the bottles were swabbed with ethanol before breaking the seals and opening the bottles. Two drops of 5% nitric acid was then added to 15mls of each water sample followed by two drops of 0.1 M silver nitrate solution. The cloudiness of the water samples treated with silver nitrate was taken to be indicative of the presence of chlorine in the water samples.

### Inactivation of chlorine in chlorinated samples

The disinfectant activity of chlorine in chlorinated samples was neutralized by the addition of 0.1 ml of 3%v/v sterile sodium thiosulphate solution to 100 ml of each chlorinated sample.

### Determination of total heterotrophic bacterial count

The enumeration of total heterotrophic bacterial count was carried out using both the serial dilution and the pour plate technique. Serial 10 fold dilutions in sterile water were carried out and 1 ml of each dilution wa*s* aseptically placed in sterile petri-dishes in triplicates. 20 ml of molten plate count agar (Oxoid) cooled to 45°C was then added to each of the plates and mixed thoroughly. The mixture was allowed to solidify and the plates incubated at 22°C and 37°C for 24–72 hours. The number of bacterial colonies were counted and reported as colony-forming units per millilitre.

### The determination of presence of coliform bacteria

The presence of coliform bacteria was determined by passing 100 ml volumes of each sample (in triplicate) through membrane filter units (Millipore) (0.45 μm pore size, 47 mm diameter). The filter membranes were placed on MacConkey agar plates and incubated at 37°C for 24 h.

### Isolation of other indicator organisms

The presence of other indicator organisms which included *Pseudomonas aeruginosa* and *Staphylococcus aureus* was determined by filtering 100 ml volumes of each sample (in triplicate) as described above. The filter membranes were placed on nutrient agar plates and incubated at 37°C for 24–48 hours. Colonies arising after incubation were observed and streaked onto fresh agar plates.

### Identification of indicator organisms

An overnight Nutrient agar culture of each isolate from each water sample was taken through Grams stain and biochemical tests for the identification of each of the organisms. The biochemical tests carried out on the Gram negative organisms included MRVP, citrate utilization, indole, growth in Triple Sugar Iron agar, and catalase. The growth characteristic of the Gram positive cocci on Mannitol Salt agar was observed and the cocci were tested for catalase and coagulase production. The Gram positive rods were observed for sporulation characteristics.

### Maintenance of strains

Distinct colonies of each isolate from the samples were stored in nutrient agar at 4°C and cryo-preserved in glycerol-nutrient broth in a deep freezer.

Statistical analysis: Descriptive statistical analysis of the data obtained from the study was carried out using the Microsoft excel 2007 version.

## Results

The total heterotrophic bacterial count of the samples at 37°C ranged between zero and 1000 cfuml^−1^. 67.4% of the water samples representing the products of 10 companies or 66.7% of the brands had heterotrophic counts within the acceptable limits. Coliforms present in 100 ml of water were detected in 16.3% of the samples and 26.7% of the bottled water brands. The coliforms detected included *Enterobacter agglomerans* from two brands and *Citrobacter freundi* from another two brands of the samples. Other indicator organisms detected included Staphylococci isolated from 27.9% of the samples (33.3% of the brands) and specifically *Staphylococcus aureus* found in four brands constituting 14% of the samples. *Pseudomonas* strains were consistently detected in consecutive batches of three brands of the water samples. The acceptability status of the bottled water samples in relation to the WHO guidelines are as shown in Table 2.

**Table 2:** **The acceptability status of the bottled water samples in relation to the WHO guidelines**

Brand	Coliforms/100 ml	TBC	Other indicators present	Acceptability
A	_	0 – 10	None	+
B	_	0 – 10	None	+
C	_	0 – 10	None	+
D	_	0 – 10	*Pseudomonas* spp.	_
E	+	11 – 100	*Staphylococcus* spp.	_
F	_	11 – 100	*Staphylococcus aureus*	_
			*Enterococcus* spp.	
G	_	101 – 500	*Pseudomonas* spp.	_
H	_	11 – 100	*Salmonella* spp.	_
I	+	11 – 100	None	_
J	+	101 – 500	*Staphylococcus* spp.	_
			*Listeria* spp.	
K	_	101 – 500	*Staphylococcus aureus*	_
L	_	11 – 100	*Staphylococcus aureus*	_
			*Listeria* spp.	
M	+	501 – 1000	*Listeria* spp.	_
N	_	501 – 1000	*Pseudomonas* spp.	_
			*Staphylococcus* spp.	
O	_	0 – 10	None	+

*TBC*, Total Bacterial Count.

## Discussion

Drinking water is not expected to be sterile therefore microorganisms may be found in potable water. There is however a limit to the number and kinds of organisms permissible in drinking water with the WHO stipulating that the heterotrophic bacteria present in bottled water should not exceed 50 cfuml^−1^ and that there should be no coliform present per 100 ml of water [16].

In this study, different batches of bottled water produced by 15 different bottling companies were investigated. These samples used were the products of large multinational water bottling companies, government and institutional bottling companies as well as the small scale bottling companies which are likely to have been attracted to the packaging of water by a combination of high demand and the possibility of making quick returns on investment [4]. From the results of this study, the three batches of samples produced by only four companies were of acceptable quality. One other company produced one acceptable batch out of the three batches sampled while the water samples from the other 10 companies were found not to be acceptable on all three sampling occasions. The acceptability of the products was determined on the bases of the total bacterial load, as well as the presence of coliforms and other indicator organisms.

On the basis of total heterotrophic bacterial load, 67.4% of the 43 water samples representing the products of 10 companies or 66.7% of the brands had heterotrophic counts within the acceptable limits for drinking or bottled water while samples from the other five water bottling companies had counts above the acceptable limits and should therefore be considered unfit for drinking. In most cases these are bacteria that are considered to be relatively harmless components of the environment and can be found on the skin, in soil and in very high numbers in food products such as fruits, vegetables, meats, cheeses, yogurt and pasteurized milk [3]. Tens of thousands to millions of such bacteria are consumed on a regular basis in these foods unnoticed [17, 18]. The presence of these organisms in large numbers even when they are non pathogenic is however of significance in immuno-compromised persons in whom they could cause opportunistic infections. Such persons include young children, the elderly, pregnant women, people with diseases such as cancer, diabetes and people on such medications as the corticosteroids which possess immunosuppressive properties [19]. It has been pointed out that high numbers of heterotrophic bacteria in bottled water might arise when unsterilized and uncapped plastic bottles are transported to the bottling plants in cardboard cartons which expose the interiors of the bottles to airborne contaminants [20]. It has also been pointed out that the presence of large numbers of heterotrophic bacteria in bottled water is an indication of poor manufacturing practices involved in the processing of such water [10] and must therefore be deemed to be unacceptable.

On the basis of the coliform count, it is required that drinking water must not contain any coliform in 100mls of water [16]. However, coliforms in 100 ml of water were detected in seven (16.3%) of the samples representing products from four (26.7%) of the producers making these brands unacceptable for drinking. The detected coliforms were from one of the three samples produced by the institutional water bottling business ventures and three of the nine small scale producers. None of the nine samples produced by the three multinational large scale producers were contaminated with coliform bacteria. Coliforms have similarly been reported to occur in bottled water sampled in Egypt and some other locations in Nigeria ([10, 12]. The presence of coliforms in bottled water samples is not a confirmation of recent fecal contamination as *E. coli*, the indicator of recent fecal contamination was not detected in any of the samples tested. Since coliforms are among the group of organisms that have been reported to have the capacity to form biofilms on the items of equipment used in the bottling process [16], they are not only indicators of problems with the quality of water source but also of possible contamination during the process of bottling water and are significant from the point of view of hygiene [21]. Some possible reasons for the presence of coliforms in bottled water samples include poor hygienic practices of the producers, poor hand hygiene, illiteracy and unhygienic practices of vendors. In a study carried out in Egypt [10] coliforms were detected in 28.6% of 84 bottled water samples examined and they were not of faecal origin as *E. coli* was absent in the samples.

On the basis of the presence of other indicator organisms, bottled water samples from 10 of the producers were found not to be acceptable for drinking. Apart from coliforms, other indicator organisms including *Staphylococcus aureus* and *Pseudomonas* species were isolated from water samples obtained from ten of the fifteen producers studied. As pointed out by Addo et al. (2009) these are common environmental contaminants which are likely to reduce the efficiency of any treatment process [22]. Pseudomonads including *Pseudomonas aeruginosa* have been shown to be persistent contaminants of water plants [23]. This organism is an opportunistic pathogen, the ingestion of which can cause infections in the immuno-compromised subjects [24]. *Pseudomonas aeruginosa* is also known for its resistance to many antimicrobial agents making the treatment of infections caused by the organism a difficult task. This is why it has been suggested that test for the presence of *Pseudomonas* species in drinking water should be used as a means of monitoring the hygienic quality of drinking water [16]. The two brands, with all batches contaminated by *Pseudomonas* species were products of two small scale producers.

Staphylococci which are the other indicator organisms detected in some of the samples were found in 11 (25.6%) of the samples and six of the brands while the species *aureus* was found in three brands. *Staphylococcus aureus* is an indicator of poor hygienic practices during water bottling [16] and is more likely to have been introduced into the samples by the personnel involved in water processing. Its presence in the water samples is suggestive of the fact that the bottled water samples in this study were contaminated not only by the factory surroundings but also by the people who came in contact with any part of the bottling procedure [25]. The use of bare hands at different stages in the production of the bottled water is a probable source of this bacterial contaminant. This is suggestive of the fact that the bottles were filled manually as is often the case with small scale producers, such as those whose samples were found to be contaminated with *S. aureus* in this study.

The deficiencies observed in these samples suggest that most of the producers of the bottled water samples examined in this study do not follow stipulated guidelines in their production processes. This is however avoidable if there is adequate control or monitoring of the practices of the bottling companies. The observation from this study is an indication of the lack of required infrastructure within the manufacturing outfits. Requirements such as clean rooms, automatic bottle filling machines, clean water source, adequately trained personnel and the need for staff supervision by personnel who are knowledgeable in the science of water purification and dispensing must be met by companies which are involved in the production of water packaged for human consumption. This implies that the capital needed by a prospective water bottling company in order to put all these things in place must be quite high. It is not surprising therefore to observe that virtually all the bottled water brands (A, B and C) produced by the large scale multinational companies were of acceptable quality. These companies are multimillion dollar companies which also produce a wide range of consumable products. They are therefore strictly regulated and their production facilities are equipped with a wide range of sophisticated equipment operated by qualified personnel and their activities supervised by adequately trained professionals. On the other hand, brands G to O were products of small scale business ventures located within Ile-Ife and its environs and all except brand O fell short of stipulated standards in terms of either the total bacterial load or the presence of coliforms and indicator organisms. Results from this study suggest that these companies have less sophisticated water bottling facilities, none to a few years of experience and most probably employ cheap labour with little or no technical knowledge in water processing. This phenomenon has been pointed out earlier by authors [26] who found out that body creams produced by large companies had better microbiological quality than creams produced by small scale producers which may not have been able to put in place the entire infrastructure needed for the production of large quantities of products with demonstrable quality.

## Conclusions

The findings from this study indicate that the water produced by the 10 institutional, medium and small scale producers ought not to be in the market for public consumption underscoring the relevance of regulatory agencies. There is a need for a rigorous inspection and follow-up of water bottling facilities so that only those companies which consistently produce water of acceptable bacteriological quality are allowed to produce water for public consumption.

## Authors’ information

OAI is a post Ph. D candidate in the Department of Pharmaceutics, Obafemi Awolowo University, Ile-Ife, Nigeria. Pharmaceutical Microbiology is her area of specialization. AL is a professor of Pharmaceutical Microbiology in the Department of Pharmaceutics of the same university.
